# Computed Tomography Assessment of Os Trigonum in the Omani Population and Its Clinical Relevance

**DOI:** 10.3390/diagnostics15030373

**Published:** 2025-02-05

**Authors:** Zahran Al Thuhli, Mohammed Al Farsi, Yasser Mahfouz, Ghassan Al Mamari, Younis Al-Mufargi, Yassine Bouchareb, Srinivasa Rao Sirasanagandla

**Affiliations:** 1 Radiology Residency, Oman Medical Specialty Board, Muscat 132, Oman; zahran.althuhli@gmail.com; 2 College of Medicine and Health Sciences, Sultan Qaboos University, Muscat 123, Oman; s141849@student.squ.edu.om (M.A.F.); s139058@student.squ.edu.om (G.A.M.); 3 Department of Radiology and Molecular Imaging, University Medical City, Sultan Qaboos University Hospital, Muscat 123, Oman; yassermahfouz@squ.edu.om; 4 Department of General Surgery, The Medical City Hospital for Military and Security Services, Muscat 123, Oman; hashyounis96@gmail.com; 5 Department of Radiology and Molecular Imaging, Sultan Qaboos University Hospital, Muscat 123, Oman; y.bouchareb@squ.edu.om; 6 Department of Human and Clinical Anatomy, College of Medicine and Health Sciences, Sultan Qaboos University, Muscat 123, Oman

**Keywords:** os trigonum, prevalence, computed tomography, anatomical variability, Oman

## Abstract

**Background**: Os trigonum (OT) is an accessory ossicle that develops from the failure of the secondary ossification center of the posterior talar process fusion. It is clinically significant due to its association with posterior ankle pain and impingement syndromes. Despite its tremendous clinical relevance, limited data exist on the frequency of OT in Middle Eastern populations. **Objectives**: This study aimed to determine the frequency, morphological variations, and dimensions of OT in Omani subjects using computed tomography (CT) imaging and to evaluate the sex and laterality differences in its occurrence. **Methods**: A retrospective cross-sectional study of 352 foot and ankle CT scans were conducted to assess the OT at Sultan Qaboos University Hospital. OT presence, dimensions, and classification along with patient demographics, including age and sex, were recorded. Descriptive statistical analysis and the chi-square test were employed to present the data. **Results**: The overall prevalence of OT was 10.2%, with a frequency of 11.2% on the left side and 8.9% on the right side. Type IIA was the most prevalent subtype in both feet (41.2% right, 44.4% left). The average minor-axis and macro-axis dimensions were 7.88 ± 2.998 mm and 10.76 ± 4.280 mm on the right side, while they were 8.06 ± 2.600 mm and 11.50 ± 4.997 mm on the left side. No statistically significant sex or laterality differences were observed with regard to the OT frequency (*p* > 0.05). **Conclusions**: This study provides the first detailed evaluation of OT in the Omani population, highlighting its frequency and morphological variability. These findings emphasize the importance of CT imaging in identifying OT and guiding clinical management. Future studies should explore OT’s clinical correlations to enhance its diagnostic and therapeutic implications.

## 1. Introduction

The os trigonum (OT), first described by Rosenmüller in 1804, is an accessory ossicle resulting from the failure of the posterolateral talar process to fuse during skeletal development [1]. Os trigonum syndrome (OTS) arises from recurrent plantar flexion stress, leading to overuse injuries of the posterior ankle. OTS is a clinical diagnosis characterized by posterior ankle discomfort, particularly during activities such as dancing en pointe, demi-pointe, or performing push-off techniques. It is most observed in ballet dancers and soccer players [2].

The OT forms due to incomplete fusion of the secondary ossification center of the posterior talar process. Embryologically, the talar body and posterior talar process develop as separate ossification centers, with the OT appearing between the ages of 7 and 13. Typically, it fuses with the talus within a year; however, in about 7% of adults, it remains unfused, occurring unilaterally or bilaterally [3]. While usually presenting as a single bone less than 1 cm in size, the OT can also appear in multiple fragments and exhibit significant variability in size and morphology [4,5]. A recent meta-analysis of 18 studies involving 17,626 ankles reported a pooled prevalence of OT at 10.3%, with no significant sex or laterality differences [6]. The OT is typically triangular but may also be rounded or ovoid [5]. Imaging, particularly through computed tomography (CT) and magnetic resonance imaging (MRI), plays a crucial role in diagnosing OTS, assessing the OT size, morphology, and clinical impact.

The morphological and dimensional variability of OT is clinically significant, influencing its functional role and association with posterior ankle impingement syndrome (PAIS). During plantar flexion, the OT or an enlarged posterior talar tubercle may impinge on adjacent soft tissues, leading to posterior ankle pain, stiffness, and swelling. Associated pathologies, such as flexor hallucis longus (FHL) tenosynovitis, are common due to the anatomical proximity of the OT to the tendon [2,7,8]. Proper classification and measurement of OT through imaging are critical for distinguishing symptomatic cases from incidental findings, guiding treatment strategies ranging from conservative management to surgical excision [2,8]. Despite its clinical relevance, there are no published studies on the prevalence of OT in Middle Eastern populations, including Oman. Furthermore, the potential sex and laterality differences in OT occurrence have been minimally explored [6]. This study aimed to determine the frequency of OT in Omani subjects using CT imaging of the foot and ankle, while also evaluating sex and laterality variations in OT occurrence.

## 2. Materials and Methods

### 2.1. Study Setting and Population

This retrospective cross-sectional study was conducted at Sultan Qaboos University Hospital (SQUH), and the study was approved by the Institutional Medical Research Ethics Committee. A total of 352 unilateral foot and ankle CT examinations of patients aged 9 years and older were reviewed. These patients were referred to the Department of Radiology and Molecular Imaging, SQUH, between September 2011 and September 2024. Patient demographics, including age, sex, and clinical indications, were systematically retrieved from electronic medical records through the institutional TRACK CARE system. The mean age of the participants was 35.85 ± 15.675 years (range 9 to 89 years). The clinical indications of study included CT scans, foot trauma, pain and swelling, foot biopsies, and diabetic foot ulcers. Patients with significant ankle joint deformities, metabolic, endocrine system disease, and talar fracture or who underwent any talus-related operation were not included in the study. Non-Omani nationality and scans presenting significant artifacts were also excluded from the study.

### 2.2. CT Acquisition Protocol

Foot and ankle CT scans were acquired for all patients using a Siemens Sensation 64, a 64-slice multidetector CT scanner, operating at a peak voltage of 100 kV and a tube current of 74 mAs. Image analysis was conducted via the Picture Archiving and Communication System (PACS) (Synapse PACS, FUJIFILM Worldwide, version 5.7.102). Images were captured with a slice thickness of 1 mm in the axial plane, with additional reconstructions in the coronal and sagittal planes.

### 2.3. Data Collection

**Figure 1:**
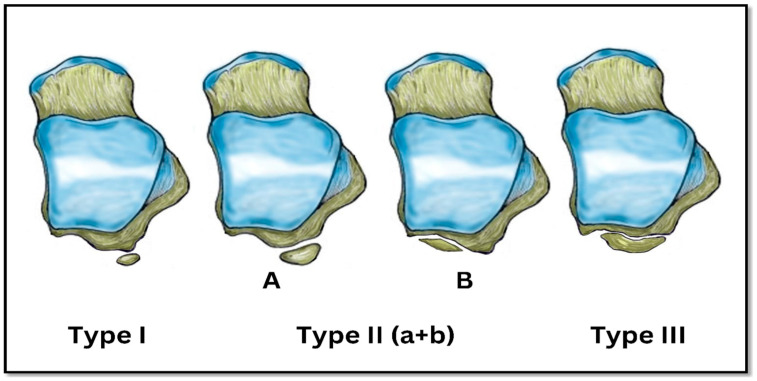
Schematic diagram showing the classification of os trigonum.

A radiology resident trained by the senior radiologist retrospectively reviewed all CT scans for OT evaluation. The observer entered each patient’s data into a Microsoft Excel spreadsheet to facilitate subsequent statistical analysis. The same observer collected data from 20% of randomly selected CT scans of included patients on two separate days for quality assurance and to avoid intra-observer errors. Additionally, a consultant radiologist reviewed randomly selected CT scans of the study sample to evaluate the collected data accuracy, particularly the OT types. The images were evaluated in a blind manner with respect to the patient characteristics, assessing the presence, size, and classification of the OT. Three types of OT were classified according to Zwiers et al., 2018 [8], type I: OT with intact lateral tubercle, type IIA: OT as part of the lateral tubercle and positioned laterally, type IIB: OT as part of the lateral tubercle and positioned medially, and type III: OT without lateral tubercle [Figure 1]. The OT dimensions were measured in the axial planes, one along its macro-axis and the other one along the minor-axis [9].

### 2.4. Statistical Analysis

The data analysis was performed using Statistical Package for the Social Sciences (SPSS, version 26.0, IBM Corporation, Armonk, NY, USA). Descriptive statistics (e.g., frequency and percentage) were applied for categorical variables to present the data. A chi-square test was used to determine the associations between the sex or side on the frequency of OT variations and the VD. A *p*-value < 0.05 was considered statistically significant.

## 3. Results

### 3.1. Demographic Characteristics

The demographic characteristics and anatomical distribution of foot and ankle CT scans among the study participants are summarized in Table 1. A total of 352 scans were included in this study, with patients’ age ranging from 9 to 89 years. The sex distribution showed that 222 subjects were male (63.1%), while 130 were female (36.9%). The anatomical distribution of foot and ankle CT imaging revealed that 54.3% of scans (n = 191) were performed on the right side, while 45.7% (n = 161) were conducted on the left side. The clinical indications of the CT scans included in the study (n = 352) were as follows: trauma-related cases were the most common indication (68.8%, n = 242), followed by pain and swelling (12.8%, n = 45), perioperative and diagnostic evaluations (11.4%, n = 40), osteoarthritis (6.0%, n = 21), infections and ulcers (0.9%, n = 3), and miscellaneous indications (0.3%, n = 1).

### 3.2. Frequency, Morphological Variations, and Dimensions of Os Trigonum

**Figure 2:**
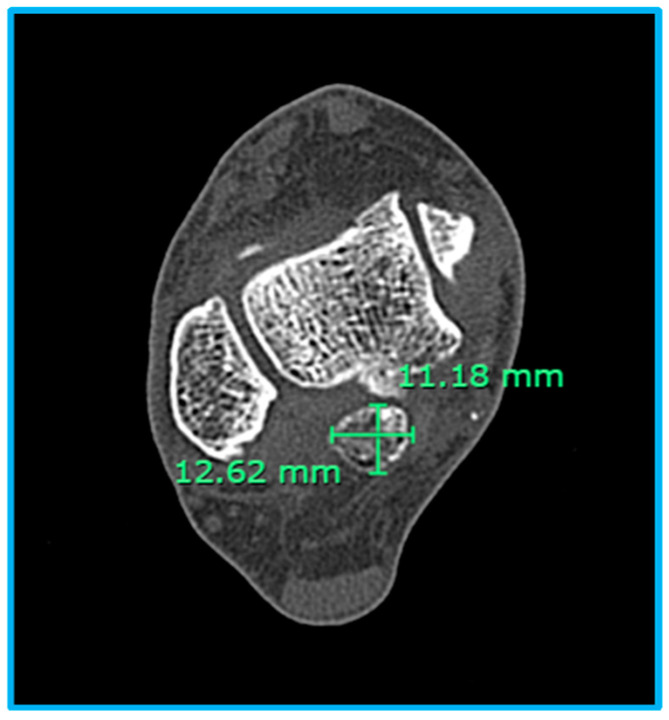
A CT axial view of the ankle demonstrates a Type I os trigonum. The os trigonum is measured, revealing a macro-axis of 12.62 mm and a minor-axis of 11.18 mm.

**Figure 3:**
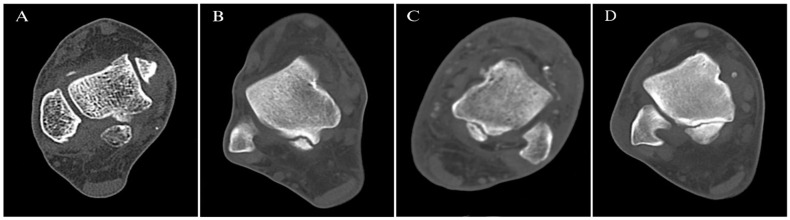
CT axial multiplanar reformatted images of the os trigonum illustrate its classification into four types: (**A**) Type I: OT with intact lateral tubercle, (**B**) Type IIA: OT as part of the lateral tubercle, positioned laterally, (**C**) Type IIB: OT as part of the lateral tubercle, positioned medially, and (**D**) Type III: OT without lateral tubercle.

A total of 191 CT scans of the right side and 161 CT scans of the left side were analyzed to evaluate the frequency, types of OT, and dimensions [Table 2]. The overall frequency of OT was 8.9% on the right side and 11.2% on the left side, while it was absent in 91.1% and 88.8% of cases, respectively. Among cases with OT, Type IIA was the most prevalent subtype, observed in 41.2% of right feet and 44.4% of left feet. Type I accounted for 35.3% of right feet and 33.3% of left feet, while Type III was present in 23.5% of right feet and 16.7% of left feet. Type IIB was rare, identified in only 5.6% of left feet. Representative CT images of OT are presented in Figure 2. The dimensions of OT were similar between the right and left sides. On the right side, the minor-axis ranged from 3 mm to 12 mm (7.88 ± 2.998 mm), and the macro-axis ranged from 3 mm to 19 mm (10.76 ± 4.280 mm). On the left side, the minor-axis ranged from 3 mm to 12 mm (8.06 ± 2.600 mm), and the macro-axis ranged from 2 mm to 24 mm (11.50 ± 4.997 mm) [Figure 3]. These results demonstrate that OT is slightly more prevalent on the left side, with Type IIA being the most common subtype in both feet.

### 3.3. Sex-Wise Frequency of Os Trigonum

The Table 3 illustrates the sex-wise frequency of OT in either side. Among males, OT was present in 11.3% of cases, while it was absent in 88.7%. Similarly, among females, OT was present in 7.7% of cases and absent in 92.3%. The chi-square test revealed no statistically significant difference in the frequency of OT between males and females (*p* = 0.280). These findings suggest that the frequency of OT does not differ significantly between males and females. The relationship between sex and the presence of OT on the right and left sides is summarized in Table 3. A total of 191 right-side and 161 left-side CT scans were analyzed. Among males, OT was present in 12 out of 118 cases (10.2%) on the right side and 13 out of 104 cases (12.5%) on the left side. Among females, OT was present in 5 out of 73 cases (6.8%) on the right side and 5 out of 57 cases (8.8%) on the left side. The chi-square tests revealed no statistically significant association between sex and the presence of OT on either side. Regarding the sex differences, a *p*-value of 0.434 was noted for the right side, while it was 0.473 for the left side. These results indicate that the distribution of OT between males and females does not differ significantly for either foot.

The frequency of OT and the mean measurements of the OT are summarized in Table 4. The descriptive statistics represent the age-wise distribution of the frequency of OT in different age groups. In the youngest age group (9–25 years), the frequency was found to be 7.4% (8/108), while in elderly individuals it was 12.5% (2/16). The mean and standard deviation values of the minor-axis and macro-axis measurements indicates the variability of the bone size in different age groups.

## 4. Discussion

The present study, to the best of our knowledge, for the first time reported the frequency of OT in the Omani population. The study results revealed a frequency of 8.9% for OT in the right foot and 11.2% in the left foot, with an overall frequency rate of 9.9%, which is consistent with the frequency spectrum reported in a recent meta-analysis. In this study, a pooled prevalence of 10.3% (95% CI: 7–14.1%) of OT was reported from 18 studies involving a total of 17,626 ankles [6]. Previous studies, using X-ray imaging, reported a frequency range of 2.3% to 9.8% [10,11,12]. However, this frequency is lower than CT-based studies, which have reported higher prevalence rates ranging between 20.5% and 32.5% [8,9]. Ethnic variability likely plays a role, as higher prevalence rates (e.g., 27.2%) have been observed in East Asian populations [9], while African and Afro-Caribbean populations exhibited lower rates (19%) [8]. The present study findings are more consistent with a study conducted in a Mediterranean population of a Turkish cohort that documented a prevalence of 9.3% [11].

In our study, OT showed a slightly higher frequency in the left foot (11.2%) compared to the right foot (8.9%), although this difference was not statistically significant. This aligns with findings from previous research, which also reported no significant preference for either side, with OT observed at 20.1% on the left and 20.8% on the right [7]. In contrast, another study found a significant difference, reporting a higher frequency of OT in the right foot (9.9%) compared to the left (8.6%) [12]. However, in support of our study results, a meta-analysis study concluded that there was no significant difference between sides with a frequency of 13.5% (95% CI: 3.9–12.1%) from 3054 right ankles and 12.8% (95% CI: 8.5–10.6%) from 3069 left ankles across five studies [6]. In another recent meta-analysis, it was mentioned that OT was present bilaterally in 32.7% of cases, indicating every third patient had OT present on both feet [13]. Since, in our study sample, CT scans were obtained for each individual with only one foot (right or left), we could not study the unilaterality and bilaterality of OT occurrence.

Our results indicate that Type IIA OT is the most prevalent subtype (41.2% in the right foot; 44.4% in the left foot), followed by Type I and Type III. Type IIB was rare, occurring in only 5.6% of left feet. This distribution aligns with prior studies that identified Type II (or equivalent subtypes) as the most frequent OT type [7,8]. Type I OT is consistently reported as the least common, with rates between 1.9% and 23% [9,10]. These patterns underscore the importance of detailed classification in clinical assessments, as type-specific morphology of OT may influence the likelihood of symptom development in patients with OT.

Regarding sexual dimorphism in the occurrence of OT, there are inconclusive reports that exist in the literature. In our study, the OT frequency was 11.3% in males and 7.7% in females, with no statistically significant difference observed (*p* = 0.280). These results are consistent with previous studies, wherein no significant differences between the sexes and OT occurrence were found [8,14]. However, certain studies indicate the possibility of sexual dimorphism. One study reported a notably higher frequency in males (13.7%) compared to females (4.3%), while another observed the opposite results, with a greater frequency in females [7,12]. The discrepancies may stem from differences in sample sizes and/or ethnic influences. In support of our study results, in the recent meta-analysis, a total of 3436 female ankles and 4574 male ankles were evaluated for sex differences [6]. The study findings reported that there are statistically significant differences. The summary of previous studies reporting the distribution of os trigonum is presented in Table 5.

OT is frequently asymptomatic but may become clinically significant in athletes, dancers, and individuals with repetitive plantarflexion, leading to PAIS and associated symptoms like pain, swelling, and stiffness [8,9]. In our study, most OT cases were incidental findings, reflecting its asymptomatic nature in the general population. The dimensions of OT identified in our cohort, with a mean macro-axis of 10.76 ± 4.28 mm in the right foot and 11.50 ± 4.997 mm in the left foot, underscore the potential for impingement in symptomatic cases, particularly when the OT exceeds 12 mm, as noted in other studies [7]. Zwiers et al. (2018) categorized the size of the OT into three groups based on axial plane measurements: smaller than 0.5 cm, between 0.5 and 1 cm, and larger than 1 cm [8]. Most OT cases in their cohort fell within the 0.5–1 cm range, highlighting significant variability in size. In our study, the mean macro-axis dimensions were 1.8 cm for the right foot and 1.2 cm for the left foot, placing most cases near the upper range of Zwiers’ second category. Additionally, Fu et al. 2019 reported that Type II OT had a significantly larger macro-axis of 8.9 ± 3.1 mm compared to other types, reflecting its potential for greater clinical impact in symptomatic cases [9]. Other study highlighted that an OT size of 9 mm or higher was sufficient to produce symptoms in pediatric populations, emphasizing the importance of dimensional thresholds in evaluating PAIS [6]. These findings align with evidence suggesting that OT sizes over 12 mm are particularly prone to impingement-related complications [6]. Variability in OT dimensions, as observed across populations, may depend on factors such as ethnic background and imaging modality. For instance, studies using CT scans have shown a higher frequency and larger size measurements compared to cadaveric studies, which may underestimate true dimensions [6,9].

It has been demonstrated that the OT prevalence increases with increasing age [13] and OT frequency differed for different age groups [13]. However, recent studies did not study age’s influence on OT prevalence [8,9]. In the present study, the OT frequency increased with age and differed in increasing age groups. Due to a small sample size, we could not perform statistical analysis. A study with large sample size exploring the role of age on OT frequency is warranted.

OT is clinically significant due to its association with posterior ankle impingement and the potential to cause chronic pain and disability if left untreated [6]. The early recognition and treatment of patients with OT are crucial to avoid long-term complications, such as chronic inflammation or tendon damage [6]. Tailored rehabilitation programs are vital, especially for athletes, to ensure a safe return to activity while minimizing the risk of recurrence. With timely and appropriate management, most patients can recover fully, but proactive strategies, including education and activity modification, remain key to reducing the burden of the condition [8,16]. Therefore, the baseline data of OT frequency and the morphological variations are clinically important.

This study adds to the understanding of OT frequency and morphological variations in the Omani population with the following limitations. Firstly, due to the retrospective design, the study included only the patients undergoing computed tomography for specific clinical indications of the ankle or foot. Secondly, the absence of clinical follow-up data limits the ability to correlate imaging findings with symptomatic presentation, impeding a comprehensive assessment of OT’s clinical implications.

## 5. Conclusions

This study’s results provide valuable insights into the frequency, morphological variations, and morphometry of OT among Omani subjects. The overall frequency of OT was found to be 9.9% with no significant side or sex differences in its occurrence. Type IIA OT was found to be the most prevalent, followed by other types. The knowledge about the prevalence, variability, and morphometry are crucial for guiding appropriate clinical management, ranging from conservative interventions to surgical excision.

## Figures and Tables

**Table 1:** Demographic characteristics.

Variable	Mean ± SD (Range)/N (%)
Age (years)	35.85 ± 15.675 (9–89)
Sex Distribution	
- Male	222 (63.1%)
- Female	130 (36.9%)
Anatomical Location of CT scans	
- Right Foot	191 (54.3%)
- Left Foot	161 (45.7%)

**Table 2:** Frequency, morphological variations, and dimensions of OT on right- and left-side CT scans (N = 352).

Category	Right Side (n = 191)	Left Side (n = 161)
Presence of Os Trigonum		
Absent	174 (91.1%)	143 (88.8%)
Present	17 (8.9%)	18 (11.2%)
Classifications of Os Trigonum		
Type I	6 (35.3%)	6 (33.3%)
Type IIA	7 (41.2%)	8 (44.4%)
Type IIB	0 (0%)	1 (5.6%)
Type III	4 (23.5%)	3 (16.7%)
Dimensions of Os Trigonum		
Minor-axis (mm)	7.88 ± 2.998 (3–12)	8.06 ± 2.600 (3–12)
Macro-axis (mm)	10.76 ± 4.280 (3–19)	11.50 ± 4.997 (2–12)

**Table 3:** Sex-wise distribution and presence of os trigonum in right and left feet.

	Right Os Trigonum			Left Os Trigonum		
Sex	Absent (n)	Present (n)	*p*-Value	Absent (n)	Present (n)	*p*-Value
Male	106	12	0.434	91	13	0.473
Female	68	5		52	5	
Total	174	17		143	18	

Note: A *p*-value < 0.05 is considered statistically significant.

**Table 4:** Frequency and mean measurements of os trigonum axes across age groups.

Variable	9–25 (n = 108)	26–45 (n = 154)	46–65 (n = 74)	>65 (n = 16)
Prevalence of Os Trigonum (%)	7.4% (8/108)	10.4% (16/154)	12.2% (9/74)	12.5% (2/16)
Right Minor-axis (mm)	8.83 ± 2.48	7.43 ± 3.46	5.67 ± 1.15	10.00
Right Macro-axis (mm)	12.83 ± 4.99	9.86 ± 4.56	9.00 ± 1.00	12.00
Left Minor-axis (mm)	6.00 ± 2.83	8.44 ± 2.70	8.00 ± 2.76	9.00
Left Macro-axis (mm)	9.00 ± 5.66	12.33 ± 3.74	9.00 ± 3.69	12.00

**Table 5:** The summary of previous studies reporting the distribution of os trigonum.

Study	Radiograph	Sample Size (Ankles/Patients)	Prevalence (%)	Male (%)	Female (%)	Type 1 (%)	Type 2 (%)	Type 3 (%)
Zwiers, Baltes, Opdam, Wiegerinck, and Van Dijk, 2018 [8]	CT	1256/628	32.5%	33.3%	31.4%	19.6%	50.3%	30.1%
Fu et al., 2019 [9]	CT	1011/586	27.2%	23.2%	13.1%	1.9%	10.5%	14.7%
Cilli and Akçaoğlu, 2005 [15]	NA	464	23.5%	23.5%	N/A	NA	NA	NA
Coskun et al., 2009 [11]	XRAY	984	2.3%	65.2%	34.7%	NA	NA	NA
Scheuermann et al., 2018 [14]	XRAY	410	11.7%	25%	75%	NA	NA	NA
Özer and Yıldırım, 2019 [16]	MRI	333	21.6%	59.7%	40.3%	NA	NA	NA
Cicek and Bankaoglu, 2020 [12]	XRAY	1088	9.3%	13.7%	4.3%	NA	NA	NA
Kalbouneh et al., 2021 [7]	CT	1478	20.5%	18.0%	23.9%	17.5%	53.5%	29.0%
Candan, Torun, and Dikici, 2022 [10]	XRAY	1651	9.8%	61.3%	38.6%	23%	62%	15%

## Data Availability

The data supporting the reported results of this study are not publicly available due to ethical and privacy restrictions. The datasets analyzed during this study are available from the corresponding author upon reasonable request.

## Abbreviations

The following abbreviations are used in this manuscript:

CT	Computed Tomography
OT	Os Trigonum
OTS	Os Trigonum Syndrome
LD	Linear Dichroism
PAIS	Posterior Ankle Impingement Syndrome
FHL	Flexor Hallucis Longus
SQUH	Sultan Qaboos University Hospital
MREC	Medical Research Ethics Committee
SPSS	Statistical Package for the Social Sciences
PACS	Picture Archiving and Communication System
CI	Confidence Interval
